# No association between preoperative physical activity level and time to return to work in patients after total hip or knee arthroplasty: A prospective cohort study

**DOI:** 10.1371/journal.pone.0221932

**Published:** 2019-09-03

**Authors:** Anton R. Boersma, Sandra Brouwer, Wendy Koolhaas, Reinoud W. Brouwer, Wierd P. Zijlstra, Jan van Beveren, Martin Stevens

**Affiliations:** 1 Department of Orthopedics, University of Groningen, University Medical Center Groningen, Groningen, The Netherlands; 2 Department of Health Sciences, Community and Occupational Medicine, University of Groningen, University Medical Center Groningen, Groningen, The Netherlands; 3 Department of Orthopedic Surgery, Martini Hospital, Groningen, Groningen, The Netherlands; 4 Department of Orthopedics, Medical Center Leeuwarden, Leeuwarden, The Netherlands; 5 Department of Orthopedics, Röpcke-Zweers Hospital, Hardenberg, The Netherlands; Linneaus University, SWEDEN

## Abstract

**Purpose:**

It is important for patients of working age to resume work after total hip or knee arthroplasty (THA/TKA). A higher preoperative level of physical activity is presumed to lead to a better or faster recovery. Aim is to examine the association between preoperative physical activity (PA) level (total and leisure-time) and time to return-to-work (RTW).

**Methods:**

A prospective multicenter survey study. Time to RTW was defined as the length of time (days) from surgery to RTW. PA level was assessed with the SQUASH questionnaire. Questionnaires were filled in before surgery and 6 weeks and 3, 6 and 12 months post-surgery. Multiple regression analyses were conducted separately for THA and TKA patients.

**Results:**

243 patients were enrolled. Median age was 56 years; 58% had undergone a THA. Median time to RTW was 85 (THA) and 93 (TKA) days. In the multiple regression analysis, neither preoperative total PA level nor leisure-time PA level were significantly associated with time to RTW.

**Conclusions:**

Preoperative physical activity level is not associated with a shorter time to RTW in either THA or TKA patients. Neither preoperative total PA level nor leisure-time PA level showed an association with time to RTW, even after adjusting for covariates.

**Trial registry:**

Dutch Trial Register: NTR3497.

## Introduction

Total hip and knee arthroplasties are proven successful in reducing pain, increasing patient mobility, and improving quality of life in patients suffering from advanced osteoarthritis (OA) [1,2]. This has resulted in a rising trend to perform total hip arthroplasty (THA) and total knee arthroplasty (TKA) in the Western world [3,4]. In the Netherlands, the incidence of THA and TKA in 1997 was 112 and 32 per 10^5^ inhabitants, respectively. In 2012 these numbers increased to 216 and 118 per 10^5^ inhabitants [5]. This rising trend fits that of other Western countries [6,7].

The incidence of total joint arthroplasties has increased not only among retirees, but also among patients of working age (<65 years) [8,9]. Main causes of the rising trend to perform THAs and TKAs in the working population are recent improvements in implant design, bearing surfaces and surgical techniques, which have led to improved outcomes, patient satisfaction and enhanced implant durability, and the delayed retirement in many Western countries, which has extended work life [10,11]. Between 1995 and 2003, the number of THA patients in the Netherlands rose by 50% and the number of TKA patients even tripled; these figures are expected to rise further [12]. For the USA, it is estimated that by 2030 up to 52% of THAs and 62% of TKAs will be performed on patients of working age [8,13]. Moreover, the epidemic of obesity has resulted in a higher prevalence of overweight workers. Overweight has been found to be an important risk factor for the development of osteoarthritis [14].

For those patients of working age, it is important to resume work after surgery [15]. Although a majority of patients of working age return to work (RTW) within one year postoperatively (68%-95% after THA; 71%-83% after TKA), long-term sickness absence is frequently reported in this period [16,17]. To prevent long-term sickness absence and accelerate the RTW process, several studies have examined factors predicting time to RTW in THA and TKA patients. Positive motivation to RTW, a higher educational level, and a higher level of physical functioning are predictors for a shorter time to RTW after THA or TKA [15,18,19]. Less pain preoperatively and having a physically demanding job were associated with a longer time to RTW [1,18].

An additional factor that could potentially have a positive influence on RTW is the concept of prehabilitation. Prehabilitation can be defined as preoperative interventions aimed at improving preoperative physical functioning in order to improve function postoperatively following THA/TKA [20] The concept of prehabilitation is based on the assumption that a higher preoperative level of physical functioning leads to a better or faster postoperative recovery [21]. In the last decade prehabilitation is increasingly being introduced in the treatment of THA and TKA patients with OA [22–24]. Within this context, several hospitals in Australia have already introduced prehabilitation in the form of preoperative physiotherapy programs for patients with OA who are on a waiting list for a THA or TKA [25]. These programs are designed to enhance physical function and minimize patient anxiety before surgery, as well as improve physical outcomes after surgery. However, a recent systematic review by Hoogeboom et al. concluded that so far it remains unconfirmed whether prehabilitation affects recovery in THA and TKA patients [23].

It would therefore be of interest to look into the effect of overall preoperative physical activity (PA) levels instead of examining the effect of rehabilitation programs specifically. PA comprises any body movement produced by the skeletal muscles that result in a substantial increase over the resting energy expenditure [26]. There are indications from the general working population that PA reduces sickness absence [27,28]. Workers with higher levels of PA were generally less likely to be absent from work because of sickness [17,19,29]. However, whether preoperative PA levels also play an important role in the RTW process has so far been scarcely investigated. Research by Hoorntje et al. [30] revealed that preoperative PA levels in patients after TKA were not associated with RTW. However, they did not look into the association between preoperative PA levels and time to RTW [30].

Aim of the current study is therefore to examine the association between the preoperative PA level and time to RTW after THA and TKA. It is hypothesized that patients who have a higher preoperative physical activity level return to work faster/earlier. As the most benefit will be expected from moderate-to-vigorous physical activities such as sports and leisure-time chores, both total and–more specifically–leisure-time PA level during time to RTW will be studied [31]. Given that rehabilitation after TKA is considered more difficult than THA [16,32], separate analyses will be conducted for each.

## Methods

### Study design and procedure

A prospective multicenter survey study was conducted among patients who underwent THA or TKA for primary OA (Dutch Trial Register; trial id: NTR3497). Patients were recruited between March 2012 and July 2014 and followed until July 2016 at the Departments of Orthopedics of the following Dutch medical centers: University Medical Center Groningen (tertiary university hospital), Martini Hospital Groningen (large teaching hospital), Medical Center Leeuwarden (large teaching hospital), and Röpcke-Zweers Hospital Hardenberg (general hospital), all in the northern Netherlands. Patients placed on a waiting list for THA or TKA were contacted by phone and invited to participate in the study. Questionnaires were mailed to be filled in at home by patients who agreed to participate. Preoperative questionnaires were filled in approximately one month before surgery. Postoperative follow-up data were collected after 6 weeks and 3, 6 and 12 months. If applicable, missing answers were added later to the questionnaire after contacting the patients by telephone. This study was approved by the Medical Ethical Board of University Medical Center Groningen (METc 2012.153).

### Participants

Patients were eligible if they: (1) were between 18–63 years of age, (2) had a paid job, (3) were scheduled to undergo THA or TKA as a result of primary osteoarthritis OA, (4) had sufficient Dutch proficiency to complete the self-report questionnaire, (5) consented to participate by filling in the questionnaire at baseline, and (6) did not meet the exclusion criterion of having undergone a unicompartmental knee arthroplasty, THA or TKA because of secondary OA or revision of a THA or TKA. A dropout was defined as a patient leaving the study preterm by not filling in the 12-month postoperative questionnaire for any reason. Most prominent reasons were non-response, re-operation/second surgery and postponement of surgery.

### Primary outcome: Time to return to work

Time to RTW was defined as length of time (days) from patients’ surgery to RTW. RTW was defined as the first time participants returned to work partially or fully after surgery. In addition, the follow-up questionnaires at 6 weeks and 3, 6 and 12 months postoperatively asked whether patients had returned to work partially or fully.

### Physical activity

PA level was assessed with the Short QUestionnaire to ASsess Health-enhancing physical activity (SQUASH) [33]. The self-assessed SQUASH questionnaire is a fairly reliable and valid tool measuring the level of PA of a healthy adult population. This has also been proven in patients after THA [34]. The SQUASH measures habitual activities during an average week for the following domains in recent months: occupation, leisure time (walking, cycling, gardening, home repairs, sports), household tasks, commuting, and other daily activities. Intensity of household tasks and activities at work and school are dichotomized into light and vigorous intensity, while time spent on these two domains is depicted in average amount of hours per week. Commuter traffic and leisure-time and sport activities are prestructured into three categories: light, moderate, and vigorous intensity. Activity scores were calculated for separate questions by adding the weekly average of total minutes per activity and multiplying it by the intensity. In this study we used the total time and leisure-time PA scores, both expressed as minutes per week. Total score was calculated by summing the activity scores of the domains. Higher scores represent a higher PA level.

### Covariates

The following covariates were assessed preoperatively, including the following items in the self-administered questionnaire: sex, age, body mass index (BMI), highest level of education (lower, secondary or higher), living with or without a partner, number of self-reported comorbidities by means of a 28-item questionnaire [35], type of arthroplasty (THA or TKA), and work demands (mainly physically demanding jobs, mainly mentally demanding jobs, or a combination of the two). Physical functioning was assessed with the Western Ontario and McMaster Universities Osteoarthritis Index (WOMAC) [36], a disease-specific questionnaire to measure self-reported constraints in physical functioning in patients with hip or knee OA [37]. The Dutch version of the WOMAC questionnaire is considered reliable and valid in patients with hip OA [38].

### Statistical analysis

Standard descriptive statistics (N, %, mean, standard deviation, median, interquartile range) were used to describe baseline characteristics of the study population. Due to the skewed outcome measure (time to RTW), data were transformed. After transformation the data was much more normally distributed and suitable to be fitted into the regression model. Regression analyses were conducted separately for the subsamples of THA and TKA patients. First, a simple linear regression analysis was performed to assess the association between covariates and time to RTW. Next, two multiple linear regression analyses were performed: one with total PA level and one with leisure-time PA level. Both analyses were adjusted for covariates used in the simple regression analysis with p<0.20. A p-value of <0.05 was considered statistically significant. Statistical analyses were performed with IBM Statistical Package for the Social Sciences (SPSS) v. 22.0 and Statistical Analysis System v. 9.4 (SAS Institute, Cary, NC, USA). In order to meet the assumptions of statistical methods, variables were transformed using the natural logarithm function.

## Results

**Fig 1:**
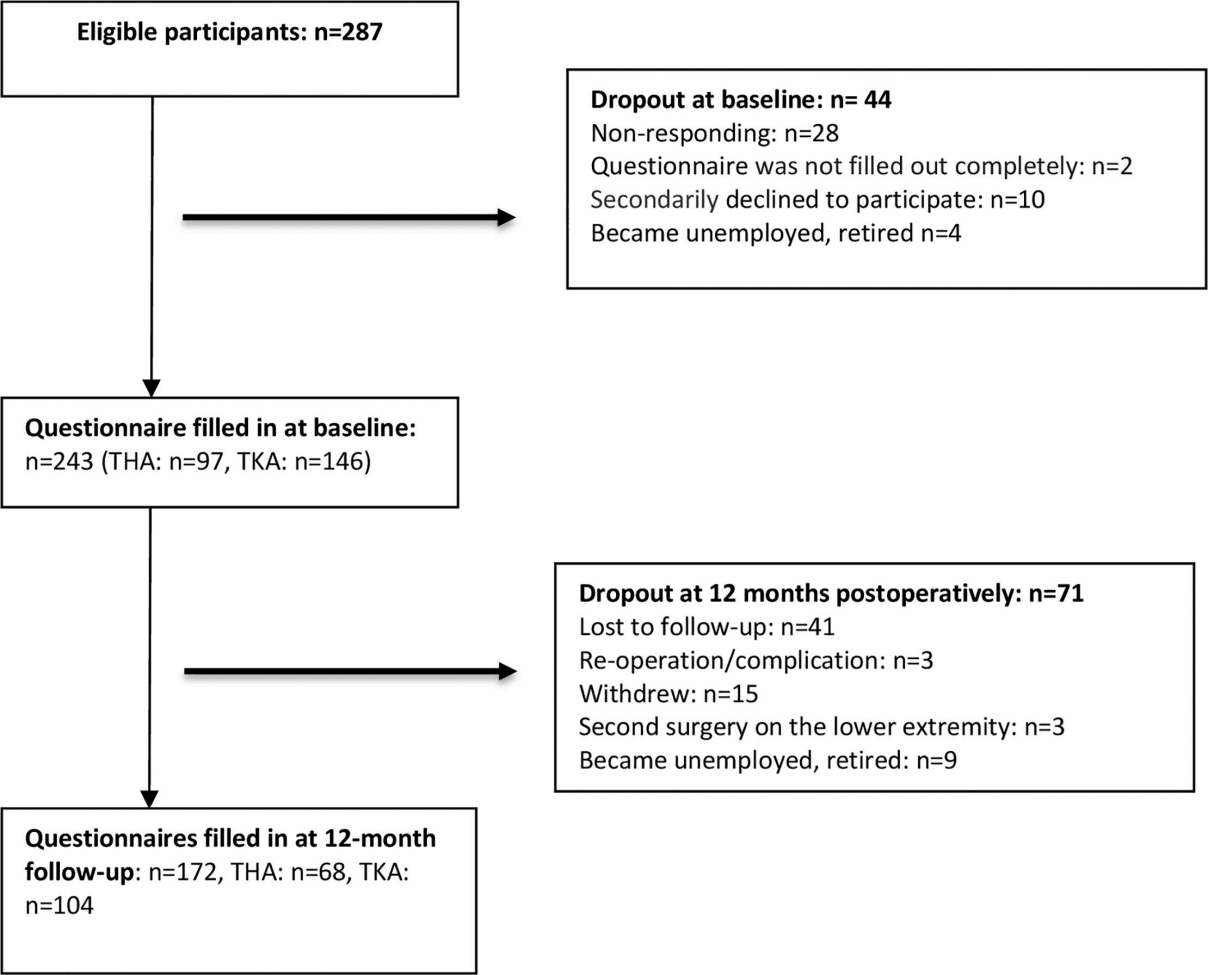
Flowchart study enrolment and follow-up.

Patients who had undergone a primary THA or TKA were considered eligible for the study. Out of 287 patients who were eligible, 243 (n = 97 THA, n = 146 TKA; response rate 85%) filled in the questionnaire at baseline (see Fig 1. Flowchart). The characteristics of the study sample are presented in Table 1. Median age of the total patient group was 56 years (interquartile range, IQR 51–59 years). The patients were predominantly female (57%), had undergone a THA (58%), and had completed lower (34%) or secondary (45%) education. Mean time to RTW of patients was 85 days (SD 69) following THA and 93 days (SD 71) following TKA.

**Table 1:** Sample characteristics.

Variable	n	Total group	THA	TKA
			n = 97	n = 146
Sex (n (%))	243			
*Female*		138 (57)	56 (58)	89 (56)
Median age (yrs (IQR))	243	56 (51–59)	56 (50–60)	56 (52–59)
Body mass index (kg/m^2^) (mean (SD))	238	28.8 (5.2)	27.3 (4.9)	29.9 (5.2)
Highest level of education (n (%))	238			
*lower (Elementary school*, *vocational education)*		80 (34)	31 (32)	49 (35)
*secondary (high school*, *intermediate vocational education)*		106 (45)	39 (41)	67 (47)
*higher (higher professional education*, *university)*		52 (22)	26 (27)	28 (18)
Partner (ref = no) (n (%))	242	225 (93)	89 (93)	136 (93)
Number of comorbidities (mean (SD))	242	2.3 (1.5)	2.1 (1.5)	2.4 (1.5)
Work demands (n (%))	237			
*physical*		71 (30)	27 (28)	44 (31)
*mental*		73 (31)	31 (32)	42 (30)
*mixed*		93 (39)	38 (40)	55 (39)
Time to return to work (days) (mean (SD))	175	89 (67)	85 (69)	93 (71)
Return to work (n (%))	175			
6 weeks				
*partial*		37 (19)	17 (21)	20 (18)
*full*		13 (7)	7 (9)	6 (6)
3 months				
*partial*		71 (36)	27 (35)	44 (36)
*full*		57 (29)	25 (32)	32 (26)
6 months				
*partial*		45 (24)	16 (21)	29 (26)
*full*		118 (63)	51 (68)	67 (59)
12 months				
*partial*		19 (10)	7 (10)	12 (11)
*full*		153 (84)	61 (84)	92 (84)
Total physical functioning score (WOMAC) (mean (SD))	242	43 (17)	42 (17)	44 (17)
Total minutes physical activity per week (SQUASH) (mean (SD))	241	3005 (1378)	3097 (1443)	2944 (1334)
Total minutes leisure-time physical activity per week (SQUASH) (mean (SD))	229	587 (768)	667 (881)	527 (677)

All numbers are represented as mean with standard deviation (SD), numbers (n) and percentages (%) or interquartile range (IQR)

Forty-four patients dropped out of the study at baseline, primarily because they were unreachable (n = 28) or declined to participate (n = 10). Follow-up data at 12 months were available for 172 (71%) of 243 patients enrolled (see Fig 1). Patients who dropped out during the study were representative of the study sample on all covariates. The majority of the total group of patients returned to work (36% part-time, 29% full-time) after 3 months, and about one-third of patients were initially employed part-time before resuming their full-time job (see Table 1).

### Association between preoperative physical activity level and time to RTW after THA

In the simple linear regression analysis no significant association was found between total or leisure-time PA levels and time to RTW after THA. Being female, higher BMI, secondary and higher education, and having primarily physically demanding or mixed work tasks all had a p-value <0.20 and were considered eligible for inclusion in the multiple regression analyses (see Table 2).

**Table 2:** Results of the simple regression analyses for physical activity and RTW, for THA and TKA patients separately.

Covariates		THA			TKA	
	B	SE	p-value*	B	SE	p-value*
Total physical activity (min/wk)	-1.85 10^−5^	6.01 10^−5^	0.759	-3.33 10^−5^	5.79 10^−5^	0.566
Leisure-time physical activity (min/wk)	-2.29 10^−5^	9.16 10^−5^	0.804	8.38 10^−5^	1.68 10^−4^	0.619
Female	0.325	0.158	**0.044**	0.182	0.144	0.209
Age	-0.003	0.013	0.817	0.020	0.014	**0.150**
Body mass index	0.035	0.019	**0.068**	0.013	0.013	0.349
Highest level of education^1^						
*secondary*	0.506	0.199	**0.013**	0.288	0.204	**0.162**
*higher*	0.404	0.187	**0.034**	0.036	0.191	0.852
Partner (ref = no)	-0.476	0.296	0.113	-0.004	0.254	0.883
Number of comorbidities	0.049	0.055	0.374	0.025	0.043	0.568
Work demands ^2^						
*physical*	0.682	0.195	**<0.001**	0.482	0.172	**0.006**
*mixed*	0.499	0.169	**0.004**	0.300	0.168	**0.078**
Total physical functioning	-0.006	0.005	0.219	-0.006	0.004	**0.126**

RTW = Return to Work; THA = Total Hip Arthroplasty; TKA = Total Knee Arthroplasty

* p ≤0.20 (in bold)

^1^ reference group = lower education

^2^ reference group = mentally demanding tasks

In the multiple regression analysis neither preoperative total nor leisure-time PA levels were significantly associated with time to RTW after THA (see Table 3). In the final model four covariates remained: being female (total PA level: B = 0.410, SE = 0.173, p = 0.021, leisure-time PA level: B = 0.409, SE = 0.178, p = 0.025), BMI (total PA level: B = 0.035, SE = 0.018, p = 0.047) having primarily physically demanding work (total PA level: B = 0.503, SE = 0.221, p = 0.026, leisure-time PA level: B = 0.572, SE = 0.227, p = 0.015), and having mixed work tasks (total PA level: B = 0.416, SE = 0.176, p = 0.022, leisure-time PA level: B = 0.406, SE = 0.182, p = 0.030) (see Table 3).

**Table 3:** Results of the multiple regression analyses for total and leisure-time PA levels and RTW, for THA and TKA patients separately.

	Influence of total PA level on time to RTW						Influence of leisure-time PA level on time to RTW					
	THA			TKA			THA			TKA		
Covariates	B	SE	p-value*	B	SE	p-value*	B	SE	p-value*	B	SE	p-value*
Total PA (min/wk)	2.54 10^−5^	5.62 10^−5^	0.654	-1.53 10^−5^	6.37 10^−5^	0.812						
Leisure-time PA (min/wk)							-9.07 10^−6^	8.74 10^−5^	0.918	2.07 10^−4^	1.58 10^−4^	0.192
Age	0.001	0.001	0.654	0.027	0.014	0.066	0.014	0.012	0.228	0.026	0.015	0.804
Female	0.410	0.173	**0.021**	0.052	0.171	0.764	0.409	0.178	**0.025**	-0.012	0.179	0.947
Partner	-0.459	0.283	0.110	0.030	0.254	0.907	-0.350	0.310	0.263	0.123	0.255	0.631
Body mass index	0.035	0.018	**0.047**	0.007	0.013	0.618	0.029	0.018	0.108	0.022	0.015	0.153
Highest level of education^1^												
*secondary*	0.239	0.214	0.269	0.012	0.234	0.966	0.220	0.221	0.323	-0.132	0.242	0.587
*higher*	0.149	0.187	0.427	-0.073	0.197	0.712	0.192	0.200	0.340	-0.093	0.202	0.648
Work demands^2^												
*physical*	0.503	0.221	**0.026**	0.499	0.193	**0.012**	0.572	0.227	**0.015**	0.552	0.193	**0.005**
*mixed*	0.416	0.176	**0.022**	0.456	0.171	**0.009**	0.406	0.182	**0.030**	0.486	0.176	**0.007**
Total physical functioning	0.003 10^−4^	0.005 10^−3^	0.553	-0.008	0.005	0.117	0.005	0.005	0.333	-0.007	0.005	0.181

PA = Physical Activity; RTW = Return to Work; THA = Total Hip Arthroplasty; TKA = Total Knee Arthroplasty

* p ≤0.05 (in bold)

^1^ reference group = lower education

^2^ reference group = mentally demanding tasks

### Association between preoperative physical activity level and time to RTW after TKA

There was no significant association between total or leisure-time PA levels and time to RTW after TKA using a simple linear regression analysis. Age, secondary education, having primarily physically or mixed demanding work and total physical functioning score had a p-value <0.20 and were considered eligible for inclusion in the multiple regression analyses (see Table 2).

In the multiple regression analysis, neither preoperative total PA level nor leisure-time PA level were significantly associated with time to RTW after TKA (see Table 3). In the final model two covariates remained: having primarily physically demanding work (total PA level: B = 0.499, SE = 0.193, p = 0.012; leisure-time PA level: B = 0.552, SE = 0.193, p = 0.005), and having mixed work tasks (total PA level: B = 0.456, SE = 0.171, p = 0.009), leisure-time PA level: B = 0.486, SE = 0.176, p = 0.007) (see Table 3).

## Discussion

This prospective, longitudinal cohort-study showed that a higher preoperative PA level is not significantly associated with shorter time to RTW in either THA or TKA patients. Neither preoperative total nor leisure-time PA levels showed an association with time to RTW, even after adjusting for several covariates. These results are in line with the study of Hoorntje et al. [30] among TKA patients, in which was concluded that preoperative PA levels are not associated with RTW, although time to RTW was not looked into. The results are also analogous to previous studies in patients with OA, in which no association was found between prehabilitation and recovery after THA or TKA [23,39–41]. Hoorntje et al. also suggested that not PA but patient beliefs and expectations are associated with return to work after TKA. Those findings are in line with a previous study of Styron et al. [18], who concluded that patient characteristics, particularly motivation, play a more important role in returning to work, as they overshadow the relatively smaller benefit of having a higher preoperative function. Unfortunately, we did not measure patient beliefs and expectations in our sample of THA and TKA patients–our focus was solely on the association between preoperative PA levels and time to RTW. It does however seem reasonable that those constructs may have played a role in our study too.

In line with two prospective longitudinal cohort studies [15,18], we found that both THA and TKA patients who had a more physically demanding job or a combination of a physically and mentally demanding job needed more time to RTW than those with a non-physically demanding job. It is reasonable to expect patients to have limited mobility in the early course of postoperative recovery and not be able to meet work demands, especially when the occupation involves exposure to risk factors such as kneeling or lifting. In our study, mean time to RTW was 12.1 weeks after THA and 13.3 weeks after TKA, which is comparable to most other studies [1,10,18,42–49]. Mean time to RTW in those studies showed a skewed distribution, which implies that most patients need more time to RTW. This may originate from several personal and environmental factors that have hardly been investigated yet. Our study showed that female gender and BMI (only in association with total PA level) were also of influence after THA. Studies specifically looking for the influence of gender and BMI on RTW are scarce, yet from former studies it is known that both female gender and a high BMI have a negative effect on functional outcome; this could influence a delayed RTW [50,51].

### Strengths and limitations

Strengths of our study are the relatively large number of patients in a prospective multicenter cohort study with a follow-up of 12 months. With this study design we aimed to assess a representative general Dutch study population. Comparable prospective studies were single-center with lower numbers of patients, resulting in lower associated levels of evidence and decreasing external validity [18,52]. Moreover, self-reported questionnaires like the SQUASH are short and easy to fill in. These can be used to assess the physical activity of patients after primary THA and TKA with minimal cost and burden to the subjects [34]. This study has some limitations. Measurements were self-reported, thus generally vulnerable to the effects of reporting bias. In general, patients tend to overestimate their PA level detected by self-reported questionnaires [53]. The SQUASH questionnaire has nonetheless been proven to be a fairly reliable tool to assess the PA behavior of patients after primary THA [34]. Still, in the study of Hoorntje et al. [30] it was suggested that the lack of association between PA level and RTW can be explained by the fact that patients who did not return to work or did so only partially overestimated their PA level. This phenomenon could also play a role in our study. Another limitation of the study that we lacked data on is whether the THA/TKA was the sole reason for being on sick leave. A final limitation was a 36% (87/259 patients) dropout rate at the 12-month follow-up. Still, patients for whom we had incomplete data were not significantly different in terms of patient characteristics than those with complete data. Those patients who dropped out are therefore not expected to influence the study outcome.

### Implications

Our findings suggest that preoperative PA level is not an important parameter for healthcare professionals to predict time to RTW after THA and TKA. A recent longitudinal study among TKA patients also found that patients that were in many ways in better physical shape did not return to work earlier than other patients [54]. To our knowledge these are the first two prospective studies to evaluate the association between PA level in daily life and time to RTW after THA and/or TKA. More studies are therefore needed to confirm our findings. The results of the current research must not be considered a pamphlet for not being physically active preoperatively either. Regular physical activity has been shown to be effective in primary and secondary prevention of several chronic health conditions and is linked with a reduction in all-cause mortality [26,55,56]. It also enhances musculoskeletal fitness.

So far, limited research is available aiming to investigate preoperative predictors on time to RTW after THA or TKA [15,16,18,19,57]. For future research, based on the results of our study and those of Styron et al. [18] and Hoorntje et al. [30] it looks justified to focus more on personal factors like motivation and patient beliefs and expectations. This is in line with previous research that also emphasizes the influence of expectations on outcomes in THA and/or TKA placement [58–60]. An integrated model of patient expectations by Laferton et al. [61] was recently presented which conceptualizes these associations between expectations and outcomes. This model could serve as a starting point to further elaborate on these associations. More insight into the associations between these preoperative predictors and outcomes is of the utmost relevance, as increasing numbers of patients are undergoing THA or TKA at a younger age and retirement age is rising in Western societies. It is therefore important to prevent sick leave and facilitate patients to reach their full retirement age in good health. Long-term sickness absence has major economic implications, which nowadays makes time to RTW an important outcome for patients, employers and the economy [62].

## Conclusions

The present study showed that preoperative PA level is not associated with time to RTW after primary THA and TKA. This applies to both preoperative total PA and leisure-time PA. Looking at clinical implications, our findings suggest that preoperative PA level is not an important parameter for healthcare professionals to predict time to RTW in working-age THA and TKA patients.

## Supporting information

S1 Appendix Database. (SAV)
